# Cationic Star Polymers Obtained by the Arm-First Approach—Influence of Arm Number and Positioning of Cationic Units on Antimicrobial Activity

**DOI:** 10.1021/acs.biomac.4c00882

**Published:** 2024-12-02

**Authors:** Sophie Laroque, Katherine E. S. Locock, Sébastien Perrier

**Affiliations:** † Department of Chemistry, University of Warwick, Gibbet Hill Road, Coventry CV4 7AL, U.K.; ‡ CSIRO Manufacturing, Clayton, Victoria 3168, Australia; § Division of Biomedical Science, Warwick Medical School, University of Warwick, Coventry CV4 7AL, U.K.; ∥ Faculty of Pharmacy and Pharmaceutical Sciences, Monash University, 381 Royal Parade, Parkville, Victoria 3052, Australia

## Abstract

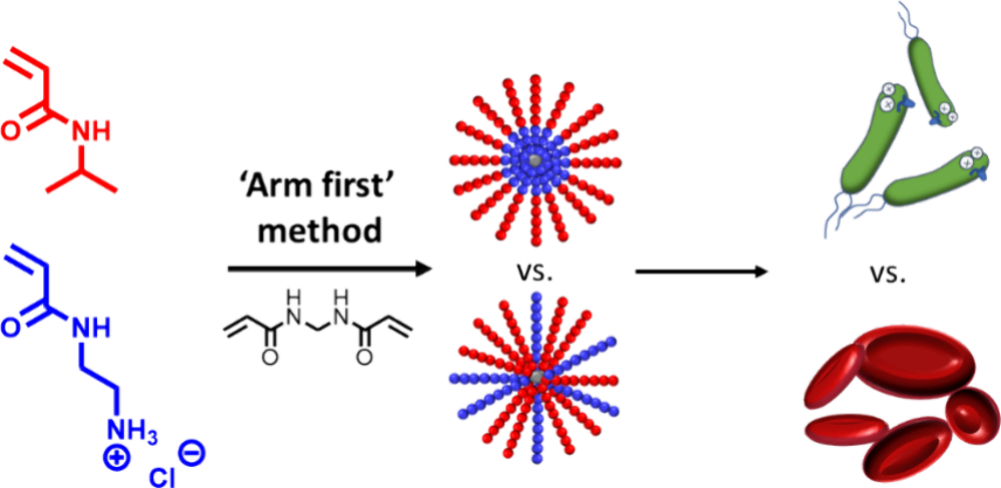

Recently, we published a study demonstrating the promising structure–activity relationship of 4-arm star polymers toward bacterial cells and biofilms. The aim of this study was to increase the number of arms to determine if this could further enhance activity via the arm-first approach, which enables access to star structures with a higher number of arms. A library of amphiphilic diblock and miktoarm star polymers was successfully synthesized, and their biological properties were assessed. The increased number of arms failed to increase activity for the diblock stars, possibly due to shielding of the cationic units located at the core from binding to the membrane, which was slightly improved for the miktoarm structures. However, the efficient synthesis of these structures shown herein could be used to synthesize star polymers with a higher cationic ratio or longer arms, thereby circumventing the limitation of reduced interaction of cationic units with the membrane.

## Introduction

Controlled polymer synthesis methods such as reversible addition–fragmentation chain transfer (RAFT) polymerization or atom transfer radical polymerization (ATRP) have made increasingly sophisticated polymer architectures such as brush polymers^1,2^ or star polymers^3−7^ available, opening up opportunities to investigate the influence of polymer architecture on biological activity. Both star and brush structures have been investigated for their antimicrobial properties and have shown great promise in improving the interaction with bacterial membranes and reducing cytotoxic effects. Star copolymers are particularly interesting, as their structures can be modulated by varying the number of arms.

**Figure 1:**
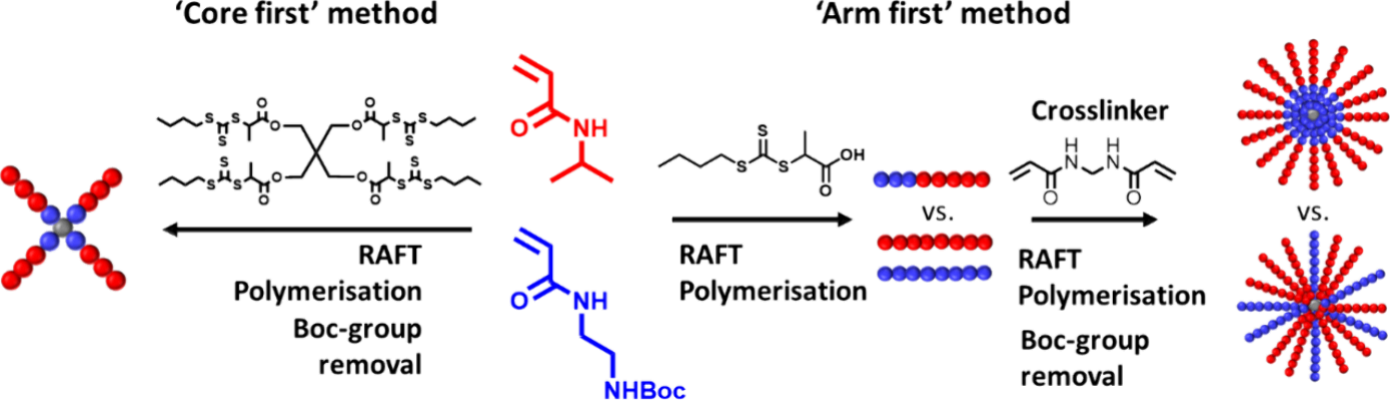
Comparison of “core-first” vs “arm-first” method with RAFT polymerization.

Shirbin et al. investigated the influence on both antimicrobial activity and hemolytic activity of the arm number and arm length in structurally nanoengineered antimicrobial peptide polymers (SNAPPs). They found that increasing the number of arms from 4 to 16 increased activity 20-fold against *E. coli*. Our group recently reported a new class of 4-arm cationic star copolymers termed synthetic star nanoengineered antimicrobial polymers (s-SNAPs),^8^ synthesized by the core-first method (Figure 1), which showed great promise as antimicrobial agents and demonstrated superior activity when compared to a linear polymer equivalent. The antimicrobial activity and hemocompatibility of these star copolymers could be tuned by changing the location of the cationic units within the arms. The most promising compound consisted of a cationic core and apolar arms, while the highest toxicity was observed for a star with an apolar core and cationic arms.

Motivated by these exciting results, we wondered if increasing the number of arms could increase antimicrobial activity further. Given that the core-first approach suffers from steric hindrance issues when increasing the number of arms growing from a core and results in poorly controlled materials with high dispersity,^9^ we adopted the arm-first approach (Figure 1). In this approach, linear copolymers, which can also be referred to as macro chain transfer agents (mCTAs), were synthesized by RAFT polymerization and chain-extended with a bis(acrylamide) cross-linker to form core-cross-linked star (CCS) polymers. This method enables the synthesis of CCS polymers with a high number of arms and good control over the length and monomer composition within the arms. This strategy also made it possible to synthesize miktoarm CCS, with arms made up of cationic or apolar homopolymers. These miktoarm CCS polymers could be seen as a compromise between the cationic units located at the core versus on the outer side of the star architecture and may therefore lead to increased activity combined with high hemocompatibility. The antimicrobial activity of the CCS polymers toward Gram-negative and Gram-positive bacteria and their hemocompatibility (hemolytic activity and hemagglutination) were investigated, and the influence of diblock CCS with a cationic core versus miktoarm CCS with cationic arms was compared.

## Experimental Section

### Materials

4,4′-Azobis(4-cyanovaleric acid) (ACVA), acryloyl chloride, Boc-anhydride (Fluka), chloroform (CHCl_3_), dimethyl sulfoxide-d_6_ (DMSO, 99.5%), diethyl ether (≥99.9%, inhibitor-free), dichloromethane (DCM), ethanol, ethyl acetate (EtOAc), ethylenediamine (99%), hexane, magnesium sulfate (MgSO_4_), Müller–Hinton Broth type II (MHB cationic-adjusted), *N*-isopropylacrylamide (NIPAM, 97%), phosphate-buffered saline (PBS) tablets, triethylamine (NEt_3_), trifluoroacetic acid (TFA), Triton-X, 1,4-dioxane (≥99%), and concanavalin A from *Canavalia ensiformis* (jack bean) were purchased from Sigma-Aldrich.

Corning Costar flat bottom cell culture plates (bottom: flat, clear, lid: with lid, polystyrene, no. of wells: 96, sterile, surface treatment: tissue culture-treated), defibrinated sheep blood, a Thermo Scientific 96-well round (U)-bottom plate, sodium chloride, and Suprasil quartz cuvettes were purchased from Fisher Scientific.

2′-Azobis[2-(2-imidazolin-2-yl)propane]dihydrochloride (VA-044) was purchased from Wako. Prewetted RC tubings (1 kDa) were purchased from Spectrum Labs. 2-((Butylthio)-carbonothioyl)thio propanoic acid (PABTC) and *N*-*tert*-butoxycarbonyl-1,2-diaminoethane (BocAEAM) were synthesized and purified according to the reported literature.^10^ The bacterial isolates used were *S. aureus* USA300, and *P. aeruginosa* PA14 was obtained by the library of Freya Harrison’s laboratory. PBS was prepared by the Media Preparation service (School of Life Sciences, Warwick University).

### Analysis

#### Size Exclusion Chromatography

The Agilent Infinity II MDS instrument was equipped with differential refractive index (DRI), viscometry (VS), dual angle light scattering (LS), and variable wavelength UV detectors. The system was equipped with 2 × PLgel Mixed D columns (300 × 7.5 mm) and a PLgel 5 μm guard column. The eluent was DMF with a 5 mmol NH_4_BF_4_ additive. Samples were run at 1 mL min^–1^ at 50 °C. Poly(methyl methacrylate) standards (Agilent EasyVials) were used for calibration ranging 955,000–550 g mol^–1^. Analyte samples were filtered through a nylon membrane with 0.22 μm pore size before injection. Respectively, the experimental molar mass (*M*_n_, SEC) and dispersity (*Đ*) values of the synthesized polymers were determined by conventional calibration and triple detection, with universal calibration using Agilent GPC/SEC software.

#### Nuclear Magnetic Resonance (NMR) Spectroscopy

^1^H NMR and ^13^C NMR APT spectra were recorded on Bruker Avance 300 and 400 spectrometers (300 and 400 MHz, respectively). Deuterated chloroform and dimethyl sulfoxide-d_6_ were used as solvents for all measurements. Data analysis was performed by using TopSpin.

#### UV–Vis Spectrometry (LCST Measurements)

Turbidity analyses for the determination of the transition temperature of each sample were performed using an Agilent Technologies Cary 100 UV–vis spectrophotometer equipped with an Agilent Technologies Cary temperature controller and an Agilent Technologies 6 × 6 multicell block Peltier. The measurements were performed using Suprasil quartz cuvettes (Hellman, 100- QS, light path = 10.00 mm) filled with 2 mg mL^–1^ solutions of each polymer in PBS. For each sample, two heating/cooling cycles between 25 and 60 °C were performed with a temperature gradient of 1 °C/min at λ = 660 nm. All data points were recorded using Cary WinUV software.

#### Dynamic Light Scattering Measurements

Size distributions and zeta potential were determined using a Litesizer 500 (Anton Paar) equipped with a 40 mW semiconductor laser diode (658 nm). Samples were prepared in PBS (pH 7.4) at 10 mg mL^–1^ and filtered through a nylon filter (0.2 μm pore size) before transferring to Suprasil quartz cuvettes (Hellman,100-QS, light path = 10.00 mm). Measurements were performed at 37 or 50 °C in a forward scattering geometry with a detection angle of 15°. Measurements for each sample were repeated three times with an automatic attenuation selection and measurement position. The results were analyzed using Anton Paar Kalliope software.

### Small-Angle Light Scattering Measurements

Small-angle X-ray scattering (SAXS) measurements were made using a Xenocs Xeuss 2.0, equipped with a microfocus Cu Kα source collimated with scatterless slits. Scattering was measured using a Pilatus 300k detector with a pixel size of 0.172 × 0.172 mm. The distance between the detector and the sample was calibrated using silver behenate (AgC_22_H_43_O_2_), giving a value of 2.491(5) m. The magnitude of the scattering vector (q) is given by where 2θ is the angle between the incident and scattered X-rays, and λ is the wavelength of the incident X-rays. This gave a q range for the detector of 0.005–0.16 Å^–1^. Samples were mounted in 1 mm borosilicate glass capillaries. Azimuthal integration of the 2D scattering data was performed, and the resulting data were corrected for absorption, sample thickness, and solvent background. All fits to the SAXS data were made using the Irena analysis macros running in Igor Pro software.^11^

### Calculations

#### Calculation of Number of Arms per Star

The average number of arms per star, *N*_arms_, can be calculated from the absolute molecular weight of the star (determined by GPC with universal calibration) and the average molecular weight of the arms (eq 1). For the calculation of the number of arms for the miktoarm stars, the molecular weight of each homopolymer was multiplied by the molar ratio used in the reaction (eq 2: 70% for pNIPAm_25_ and 30% for pBocAEAm_12.5/25_).
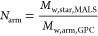
1
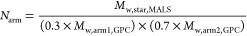
2

#### Calculation of Percentage of Arm Incorporation

The arm incorporation can be calculated by deconvolution of the multimodal SEC traces with eq 3 below, where ∫Air_Star_ is the integration of the SEC signal corresponding to the star peak, and ∫Air_Arm_ is the signal corresponding to the arms that were not cross-linked:
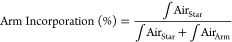
3

#### Calculation of Molecular Weight of Deprotected Star Polymers


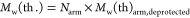
4

### Monomer Synthesis (BocAEAm)

#### Synthesis of *N*-Butoxycarbonyl-1,2-diaminoethane

A solution of ethylenediamine (26.7 mL, 400 mmol, 1 equiv) in 400 mL of DCM was added to a 1 L round-bottom flask fitted with a pressure-equalizing dropping funnel. After the solution was cooled to 0 °C with an ice-bath, a solution of di*tert*-butyl dicarbonate (8.73 g, 40.0 mmol, 0.1 equiv) in 200 mL of DCM was added dropwise over 2 h under stirring. The mixture was then allowed to warm to room temperature and left stirring overnight. The solvent was removed by rotary evaporation, and 100 mL of water was added to the residue. The white precipitate was removed by filtration, and the filtrate was saturated with sodium chloride and extracted with ethyl acetate (3 × 60 mL). The combined organic layers were dried over sodium sulfate, filtered, and the solvent was removed by rotary evaporation to yield a pale oil, identified as N*-tert-*butoxycarbonyl-1,2-diaminoethane (5.30 g, 32.0 mmol, 82% yield).

^1^H NMR (CDCl_3_): δ = 5.10 (s, 1H, CH_2_–N**H**_**2**_); 3.14 (m, 2H, C**H**_**2**_–NH), 2.78 (m, 2H, C**H**_**2**_–NH_2_), 1.37 (s, 9H,C–(C**H**_**3**_)_3_) ppm.

#### Synthesis of *N*-*t*-Butoxycarbonyl-*N*′-acryloyl-1,2-diaminoethane

*N-tert-*butoxycarbonyl-1,2-diaminoethane (5.30 g, 32.0 mmol, 1 equiv) and triethylamine (3.32 mL, 20.0 mmol, 0.7 equiv) were dissolved in 40 mL of chloroform in a 100 mL round-bottom flask fitted with a pressure-equalizing dropping funnel and cooled to 0 °C with an ice-bath while stirring. Acryloyl chloride (3.05 mL, 40.0 mmol, 1.2 equiv) was dissolved with 60 mL chloroform and added dropwise over 1.5 h while stirring. After the addition, the mixture was allowed to warm to room temperature and left stirring for 1 h. The solvent was removed under reduced pressure and dissolved in a minimum amount of chloroform. The solution was washed with 40 mL of water, which was then extracted with chloroform (3 × 60 mL). After drying over sodium sulfate, the filtration solvent was removed by rotary evaporation to yield a white powder, identified as N*-tert-*butoxycarbonyl-1,2-diaminoethane (6.20 g, 28.0 mmol, 88% yield).

^1^H NMR (CDCl_3_): δ = 6.28 (d, 1H, CH=C**H**_**2**_), 6.11 (q, 1H, C**H**=CH_2_), 5.66 (d, 1H, CH=C**H**_**2**_), 3.46 (m, 2H, NH–C**H**_**2**_), 3.35 (m, 2H, O–C**H**_**2**_), 1.46 (s, 9H, C–(C**H**_**3**_)_3_) ppm.

#### General Procedure of RAFT Polymerization Linear Copolymers

The monomer(s), *N*-isopropylacrylamide (NIPAm) and Boc-amino ethyl acrylamide (BocAEAm); the PABTC chain transfer agent, 4,4′-azobis(4-cyanovaleric acid) (ACVA); and dioxane were added to a 5 mL glass vial equipped with a rubber septum to obtain a solution with a total monomer concentration of 2 mol L^–1^. The solution was degassed with nitrogen for 15 min, and the reaction was heated in an oil bath to 85 °C. After 1.5 h, the vial was removed from the oil bath, and the reaction quenched by exposure to oxygen. Polymers were then precipitated in diethyl ether.

#### General Procedure of RAFT Polymerization for Core-Cross-Linked Star Polymers

Different ratios of linear copolymer(s), *N*,*N*′-methylenebis(acrylamide), 2′-azobis[2-(2-imidazolin-2-yl)propane]dihydrochloride (VA-044), and dioxane/water (80/20) were added to a 7 mL glass vial equipped with a rubber septum to obtain a solution with a final concentration of 0.2 mol L^–1^. The solution was degassed with nitrogen for 15 min, and the reaction was heated in an oil bath to 70 °C. After 2 h, the vial was removed from the oil bath, and the reaction quenched by exposure to oxygen. Polymers were precipitated in diethyl ether and dried under vacuum.

#### General Procedure for the Deprotection of Boc-Protected Polymers

The polymers were dissolved in 1.5 mL of DCM and 1.5 mL of TFA and stirred overnight at room temperature. The TFA was removed by precipitation in diethyl ether. Subsequently the polymers were dissolved in 10 mL of deionized water and dialyzed (MWCO 10 kDa) against an aqueous solution of sodium chloride with three water changes every 3 h, followed by a dialysis against water with three water changes every 3 h. Finally, the polymers were freeze-dried to remove water.

### In Vitro Assays

#### Minimum Inhibitory Concentration Assay

Minimum inhibitory concentrations (MICs) were determined according to the standard Clinical Laboratory Standards Institute (CLSI) broth microdilution method (M07-A9-2012).^12^ A single colony of bacteria in agar plates was chosen and dissolved in a fresh cationic-adjusted Mueller–Hinton broth (caMHB). The concentration of bacterial cells was adjusted by measuring the optical density at 600 nm (OD_600_) to obtain a McFarland standard of 0.5 to reach a bacterial concentration of approximately 1 × 10^8^ colony forming units per mL (CFUs mL^–1^). The solution was further diluted 100-fold to obtain a concentration of 1 × 10^6^ CFU mL^–1^. Polymers were dissolved in respective media, and 50 μL of each polymer solution was added to microwells, followed by the addition of the same volume of bacterial suspension, resulting in a final bacterial density of 5 × 10^5^ CFU mL^–1^. The microwell plates were incubated at 37 °C for 18 h. The growth was evaluated by the addition of 10 μL of resazurin dye of each well, leading to a final concentration of 0.5 mg mL^–1^ The plates were incubated for 30 min at 37 °C, and a noticeable change of color could be observed where bacteria cells grew (pink color) and nondetectable growth (blue color). Resazurin was prepared at 0 5 mg mL^–1^ stock in PBS, and the solution was filter-sterilized (0.22 μm filter). The solution was stored at 4 °C and covered in foil for a maximum of 2 weeks after preparation. The protocol was followed as described before by Elshikh et al.^13^

#### Hemolysis Assay

Sheep red blood cells (RBCs) were prepared by washing with PBS via centrifugation until the supernatant was clear. Polymers were dissolved in PBS up to 512 μg mL^–1^ as the highest concentration. A solution of 1% Triton X-100 was used as a positive control, and a solution of PBS was used as a negative control. A 100 μL aliquot of 6% (v/v) RBCs in PBS was added to each well of a 96-well plate. Then, 100 μL of each polymer solution was added to make up a total volume of 200 μL and was mixed before being incubated at 37 °C for 2 h. The 96-well plates were centrifuged at 600 × *g* for 10 min, and 100 μL of the supernatant was transferred to a new plate. The absorbance at 540 nm was measured and normalized with the positive and negative controls. The positive control (Triton X-100) was used as 100% cell lysis, and the negative control (PBS) was used as 0%.

#### Hemagglutination Assay

Sheep red blood cells (RBCs) were prepared by washing with PBS via centrifugation until the supernatant was clear. A 50 μL aliquot of 6% (v/v) RBCs in PBS was added to each well of a “U”-bottom 96-well microplate. Polymers were dissolved in PBS up to 512 μg mL^–1^ as the highest concentration. Concanavalin A (0.5 mg mL^–1^) solution was used as a positive control, and PBS was used as a negative control. Then, 50 μL of each polymer solution was added to make up a total volume of 100 μL and was mixed before being incubated at 37 °C for 1 h. After the incubation period, hemagglutination was assessed by comparing visually the treatment wells with the control wells.^14^ For the light microscopy images, the assay was performed in a 24-well plate only at the highest polymer concentration and directly imaged after incubation.

## Results and Discussion

### Linear Polymer and CCS Polymer Synthesis

Acrylamide derivatives were chosen as monomers since they enable the fast synthesis of complex and well-defined materials with a wide range of functional groups and are not prone to hydrolytic degradation in aqueous solution, which is of great importance in investigating their biological applications.^15^ The chain transfer agent (CTA), propanoic acid butyl trithiocarbonate (PABTC), was utilized, as it has been shown to mediate well-controlled RAFT polymerization of acrylamides,^8,10,16,17^ and the short alkyl Z-group minimizes potential solubility issues in aqueous solution, as well as steric hindrance during star polymer formation.

*N-*isopropylacrylamide (NIPAm) and amino ethyl acrylamide (AEAm) were chosen as monomers as the apolar and cationic components, respectively, based on the promising antimicrobial activity and biocompatibility of their combination found in our previous work.^8,16^ The cationic AEAm monomer was protected with a Boc-group during RAFT polymerization to prevent side reactions such as aminolysis and Michael addition and is removed postpolymerization by trifluoroacetic acid (TFA) treatment. p(NIPAm) is a thermoresponsive polymer with an LCST of approximately 32 °C and is classified as a hydrophilic monomer. However, the isopropyl side-chain is apolar, and when copolymerized with a cationic monomer, it confers an overall amphiphilic character to the final copolymers and could therefore be defined as an apolar monomer.^18^ Furthermore, the resulting polymer is typically water-soluble,^19^ although it depends on the molecular weight, comonomers, polymer architecture, and temperature.^10,16,17,20^ The LCST of p(NIPAm) can, for example, be increased through copolymerization with hydrophilic or cationic monomers, leading to water-soluble polymers at physiological temperatures of 37 °C.^20^ We therefore refer to it as “apolar” within the context of this study.

The ratio of the two monomers was kept at a 30% ratio of AEAm to 70% NIPAm throughout the polymer library. For miktoarm CCS polymers made up of homopolymer mCTA, the ratio of cationic arms was kept at 30% as well. This is based on a study performed by Kuroki et al.^10^ wherein a 30% cationic ratio resulted in a good balance between high antimicrobial activity and low cytotoxicity. For the synthesis of the CCS polymers, the bifunctional monomer, *N*,*N*-methylene bis(acrylamide) (MBAm), was utilized. This cross-linker has yielded well-defined CCS polymers with acrylamides in previous studies conducted by Bray et al.^21^ and Burgevin et al.^22^ with high incorporation of arms and low dispersities.

**Figure 2:**
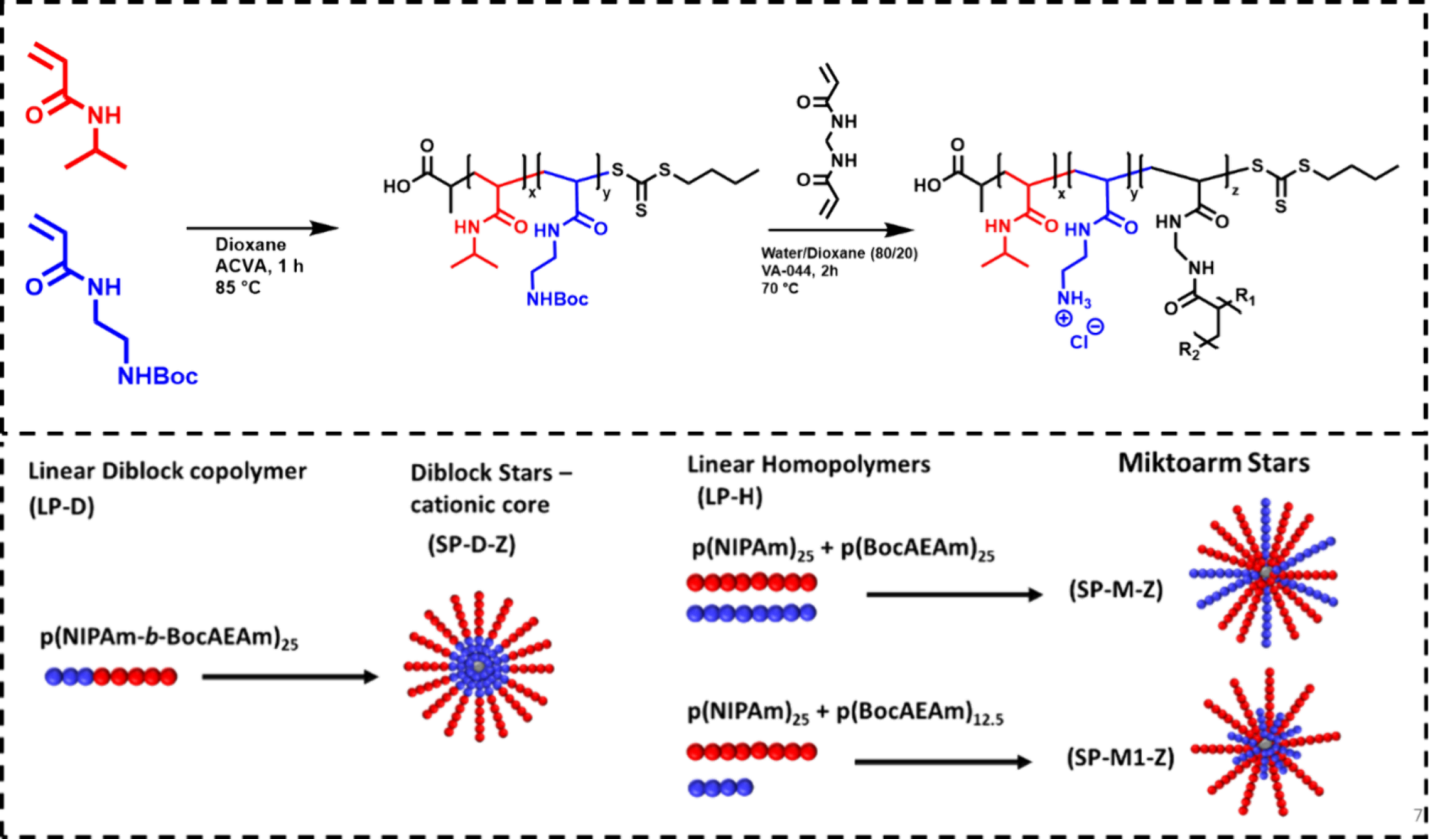
Planned synthesis and nomenclature of the “arm-first” core-cross-linked star (CCS) copolymer library. Linear polymers are labeled LP-X (LP = linear polymer, four different polymers numbered X = 1–4) and star polymers SP-D/M-Z (SP = star polymer, D = diblockarm, M = miktoarm, Z = DP of cross-linker).

The aim of this study was to synthesize a total of 10 polymers (Figure 2), four linear polymers, and six CCS polymers to investigate structural effects on biological properties. The linear diblock copolymer, p(NIPAM-*b*-BocAEAm)_25_ (**LP-1**), was core-cross-linked to form a diblock CCS (**SP-D-Z**) with a cationic core. Two homopolymers, p(NIPAM)_25_ (**LP-2**) and p(BocAEAM)_25_ (**LP-3**), were core-cross-linked to form a miktoarm CCS (**SP-M-Z**). Finally, **LP-2err** was core-cross-linked with a shorter homopolymer p(BocAEAm)_12.5_ (**LP-4**) to yield a miktoarm CCS with shorter cationic arms (**SP-M1-Z**). This miktoarm star was synthesized to mimic the concept of a cationic core with apolar arms but with a potentially higher exposure of the cationic units to the bacterial membrane, which could increase binding and insertion and therefore antimicrobial activity.

**Figure 3:**
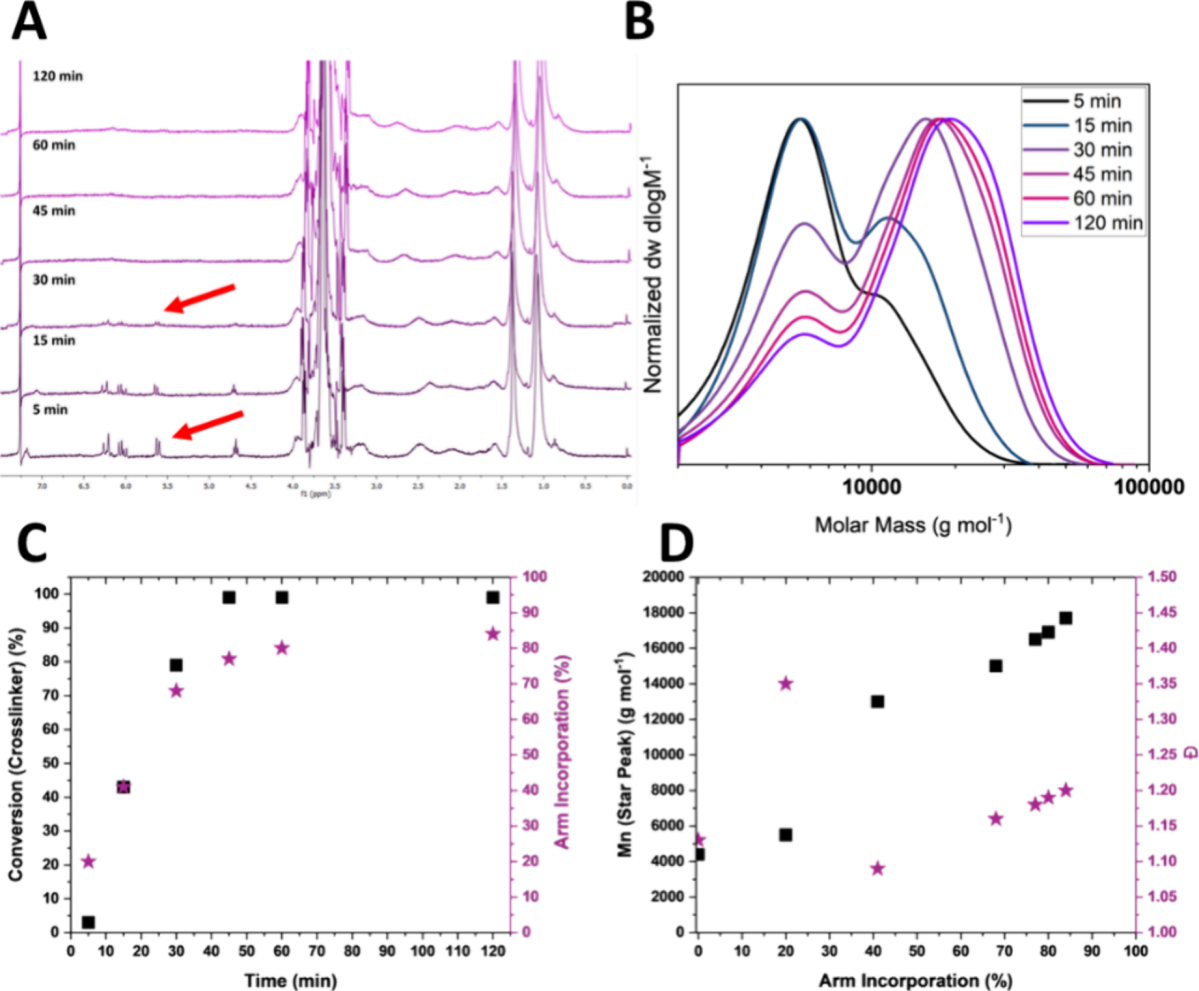
Kinetic experiment of CCS polymer synthesis. (A) ^1^H NMR spectra of time points (400 MHz, CDCl_3_). (B) SEC traces of CCS polymers (PMMA Standard, DMF). (C) Time plotted vs arm and cross-linker conversion. (D) Arm incorporation plotted vs molecular weight (*M*_n_) and dispersity.

The linear copolymers were successfully synthesized according to previously optimized protocols.^8^ In order to optimize the CCS polymer synthesis, a kinetic experiment was performed with LP-1, with dioxane as the solvent and a CL/mCTA ratio of 3. After 45 min, 99% of the cross-linker was consumed according to ^1^H NMR spectroscopy (Figure 3 and Table 2-S-4), and arm incorporation reached 77%. This was further increased to 84% after 2 h, and dispersity remained below 1.3 throughout the reaction for the CCS polymer peak (Figure 3D). Therefore, it was decided to leave the reaction running for 2 h to maximize arm incorporation. A 100% incorporation of the mCTA could not be reached, which is likely due to initiator-derived dead chains^23^ remaining from the synthesis of the initial mCTA, which cannot be reinitiated. These results are in agreement with the published literature on the arm-first CCS copolymer synthesis by RAFT polymerization.^24^

**Figure 4:**
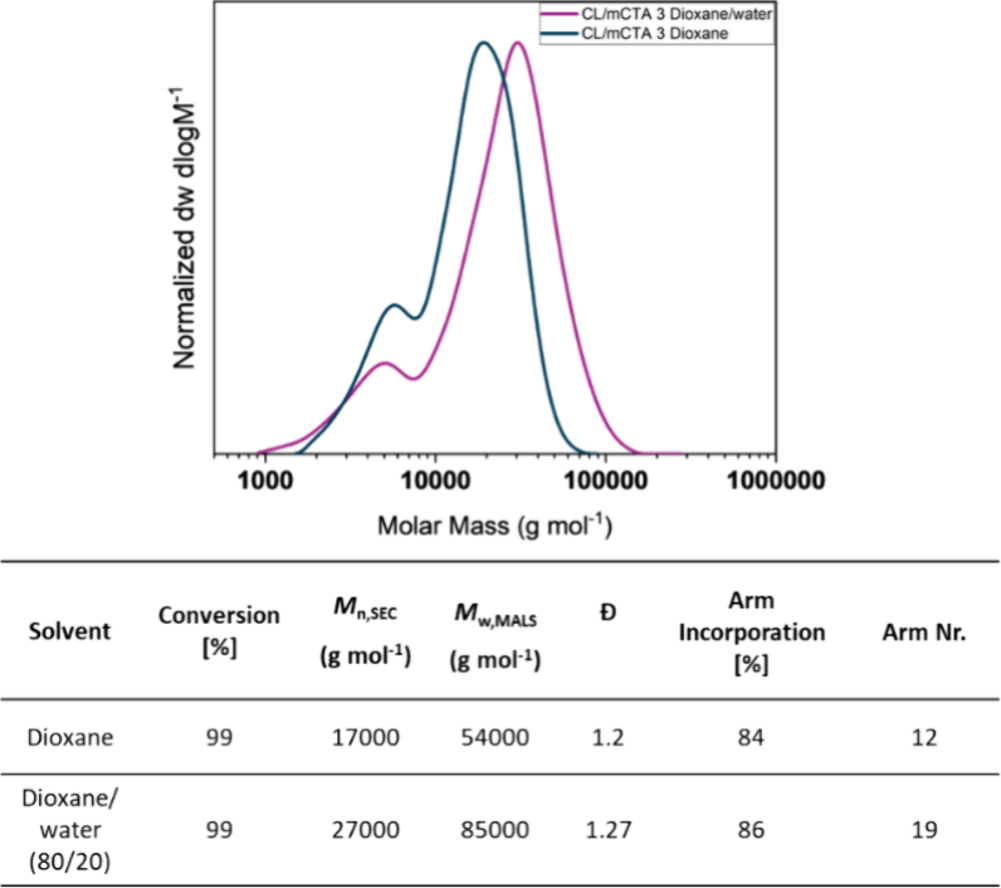
SEC traces and SEC (DMF GPC, PMMA Standard) and ^1^H NMR (300 MHz, CDCl_3_) results of CCS polymerization with two different solvents.

Solubility issues were encountered with the cross-linker (MBAm) when using 100% dioxane as the solvent, whereas the cross-linker only fully dissolved upon heating the vial in the oil bath. This led to inconsistent results especially when upscaling the reaction; therefore, a different solvent system was tested by adding 20% water to the mixture. This led to an increased solubility of MBAm; however, the dispersity slightly increased as a result (Figure 4). No turbidity was observed throughout the reaction, demonstrating that the use of water as a cosolvent with dioxane increased the LCST of pNIPAm far above the reaction temperature of 70 °C.

**Figure 5:**
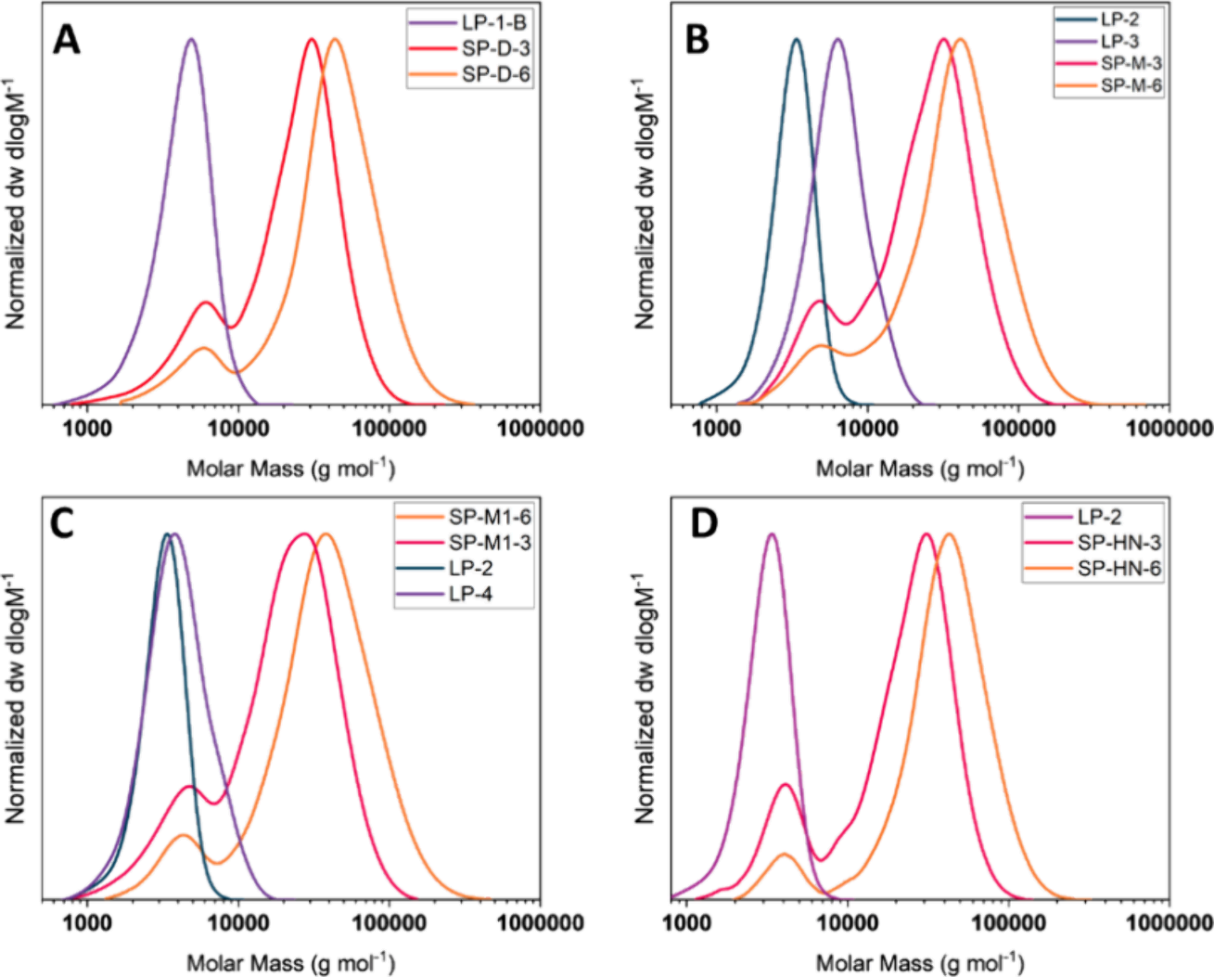
SEC traces of linear and star polymers (DMF, PMMA Standard), (A) diblock CCS, (B) miktoarm CCS with equal arm length, (C) miktoarm CCS with shorter cationic arms, and (D) homopolymer CCS with pNIPAm_25_ arms.

Finally, the MBAm to mCTA ratio was varied from 3 to 4.5, 6, and 9 to establish if this could enable us to vary the number of arms within the stars. For a ratio of MBAm/mCTA of 9, a gel was obtained, which could not be analyzed by GPC. With a MBAm/mCTA ratio of 3, an average number of 16 arms was obtained, which increased to 22 and 29 with ratios of 4.5 and 6, respectively. Dispersity values increased with an increased cross-linker ratio, likely due to star–star coupling, although they remained below 1.4. Considering the small relative difference between 20 and 23 arms, we focused the study on stars with ratios of 3 and 6, resulting in a total number of six star polymers (Table 1 and Figure 5).

**Table 1:**
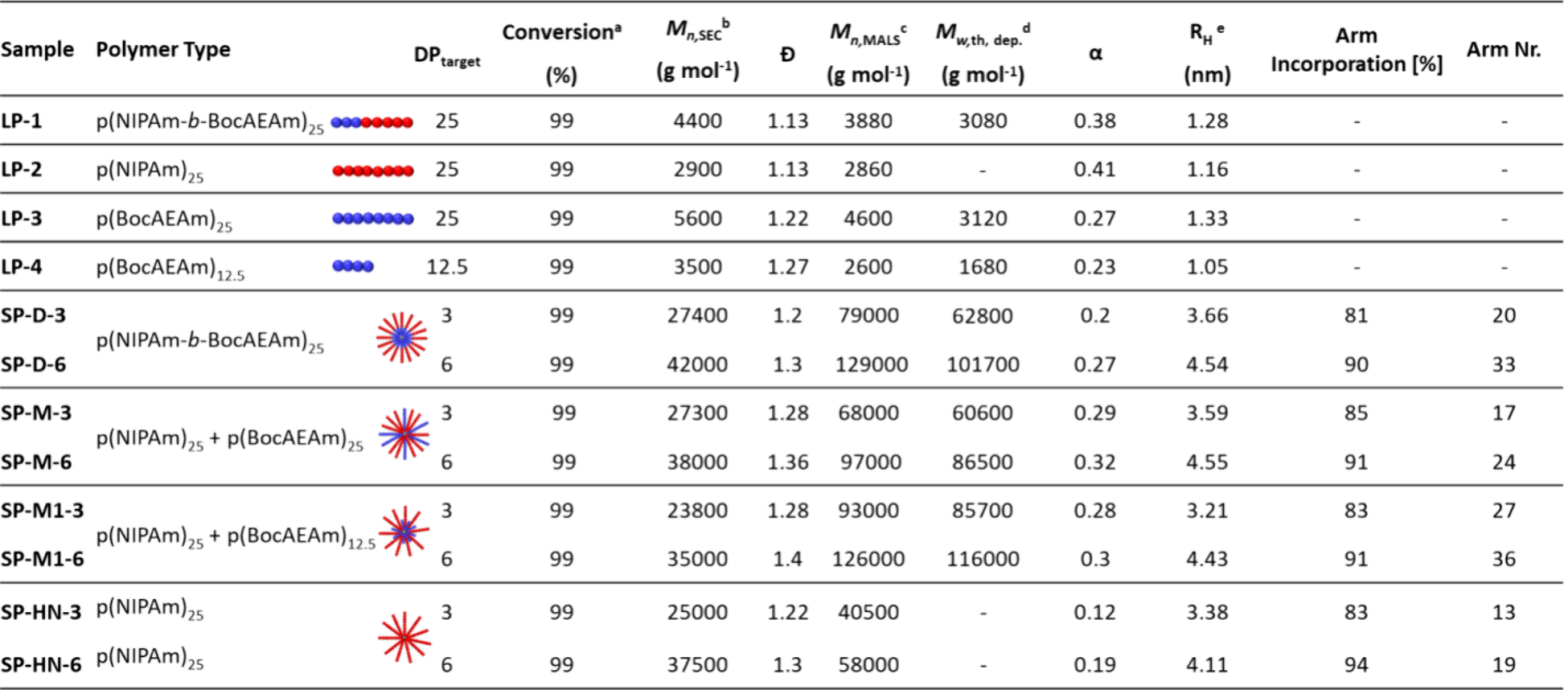
SEC (DMF GPC, PMMA Standard) and ^1^H NMR (300 MHz, CDCl_3_) Results for Linear and Star Copolymer Compounds

a Monomer (NIPAm, BocAEAm or MBAm) conversion determined by ^1^H NMR.

b Calculated based on conventional PMMA column calibration.

c Obtained through triple detection SEC with universal calibration. α = Kuhn–Mark–Houwink–Sakurada parameter from a viscometry detector.

d Theoretical molecular weight of deprotected cationic polymers.

e Hydrodynamic radius (*R*_H_) determined from a viscometry detector.

The polymers were analyzed by ^1^H NMR spectroscopy and DMF SEC. The arm number average for each star was calculated according to eq 2 (see Experimental Section). The increase of arm number with an increased cross-linker ratio from 3 to 6 appears consistent for the two SP-D and SP-M stars. For the SP-M1 stars, the overall number of arms is higher, which could be due to the smaller size of the BocAEAm mCTA, resulting in increased incorporation. The dispersities of the miktoarm stars SP-M and SP-M1 are slightly increased compared to SP-D, which is expected, as two different homopolymers are cross-linked, leading to a distribution of stars with varying ratios of pBocAEAm_12.5/25_ and pNIPAm_25_ arms.

The alpha values obtained from Mark–Houwink plots by SEC analysis with universal calibration decrease for the star polymers (0.12–0.32) in comparison to their linear precursors (0.38/0.41). Alpha values describe the relationship between intrinsic viscosity and molecular weight. A decrease in alpha can therefore also indicate branching, which usually results in a more compact structure in solution, and is therefore lowered for star polymer structures. Interestingly, the linear BocAEAM_*x*_ polymers LP-3 and LP-4 also exhibit alpha values below 0.3, which could be attributed to decreased solubility in the DMF solvent, reducing the increase of viscosity along with molecular weight.

**Figure 6:**
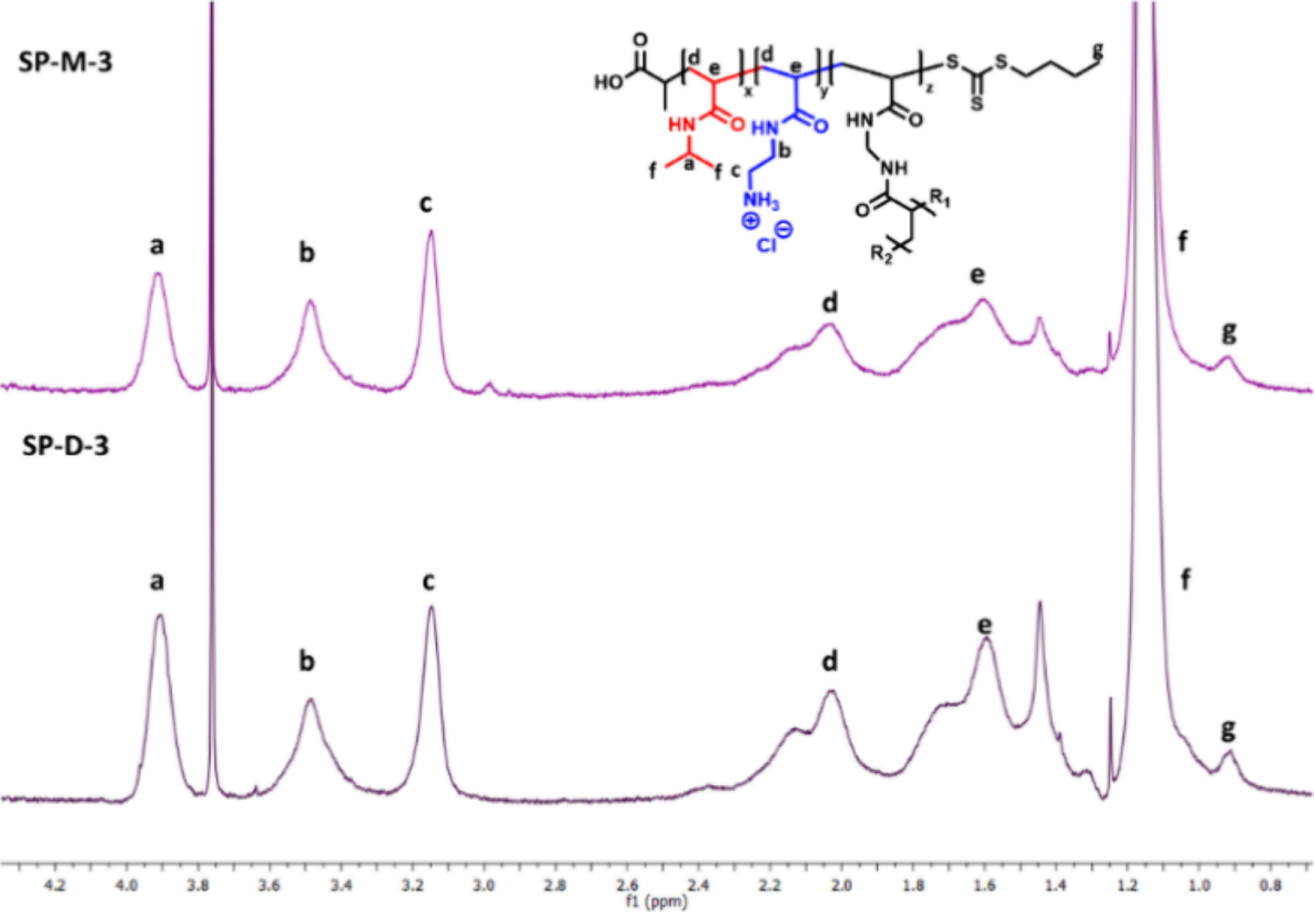
^1^H NMR Spectra of SP-D-3 and SP-M-3 (deprotected) in D_2_O.

Through integration of the ^1^H NMR signals (Figure 6) of the pNIPAm block (CH, 3.9 ppm) and the pAEAm block (CH_2_, 3.1 and 3.5 ppm), it was determined that the cationic ratio for all four miktoarm stars was kept at approximately 30% and is therefore comparable to the cationic ratio in the SP-D stars.

Verifying through analytical methods that the miktoarm stars are in fact composed of a mix of pNIPAm and pAEAm arms is very difficult. In SEC measurements, a mixture of two star homopolymers would have a similar hydrodynamic volume as a mixed-arm star and can therefore not be used to determine the arm ratio within miktoarm stars. However, as both mCTA chains used are made of the same monomer class and have similar hydrodynamic volumes (especially for the SP-M1 stars), it is reasonable to assume that homogeneous miktoarm stars were formed. Furthermore, LP-2 and LP-3, which were cross-linked to form SP-M stars, were mixed together (in a 70/30 ratio) and measured by DMF SEC, and the resulting SEC trace matches the trace of the leftover mCTA chains in the star polymer sample, showing that both mCTAs were likely consumed equally (Figure S4).

#### Physiochemical Properties of CCS Polymers

PNIPAm is known to exhibit an LCST around 32 °C, which can be varied through a multitude of factors such as molecular weight, architecture, comonomers, and even the RAFT agent.^20,25^ Therefore, the LCST behavior of the CCS polymers, dissolved at a concentration of 1 mg mL^–1^ in PBS, was investigated by ultraviolet–visible (UV–vis) measurements within a temperature range of 25–60 °C in one heating–cooling cycle. Furthermore, the LCST behavior allows us to investigate if the miktoarm stars are heterogeneous by comparing the LCST behavior of the cationic miktoarm and diblock stars to homoarm stars made up of pNIPAM_25_. These homopolymer stars were synthesized by cross-linking LP-2 at MBAm/mCTA ratios of 3 and 6, referred to as SP-H-3 and SP-H-6, respectively (Figure 5 and Table 1). The arm number for these stars was slightly lower; therefore, the molecular weight for SP-H-6 (58,000 g mol^–1^) is more comparable to SP-M-3 (68,000 g mol^–1^/60,600 g mol^–1^ upon deprotection).

In order to obtain the cationic star polymers, all six Boc-protected star polymers were deprotected with TFA and dialyzed to remove the remaining mCTA chains. The SP-M1-6 polymer could not be analyzed further postdeprotection, as it would not dissolve in aqueous solution. Successful removal of the Boc-group for this polymer was shown in ^1^H NMR spectroscopy (Figure S2) after deprotection. However, the polymer was insoluble in water or PBS and appeared to form visible aggregates. This could be due to the much higher number of arms per star (Table 1) compared to all other miktoarm stars, resulting in aggregation at room temperature. The LCST behavior of the remaining five cationic CCS polymers and two homopolymer CCS was analyzed through measuring transmittance with UV–vis spectroscopy of polymer solutions in PBS at a concentration of 2 mg mL^–1^.

**Figure 7:**
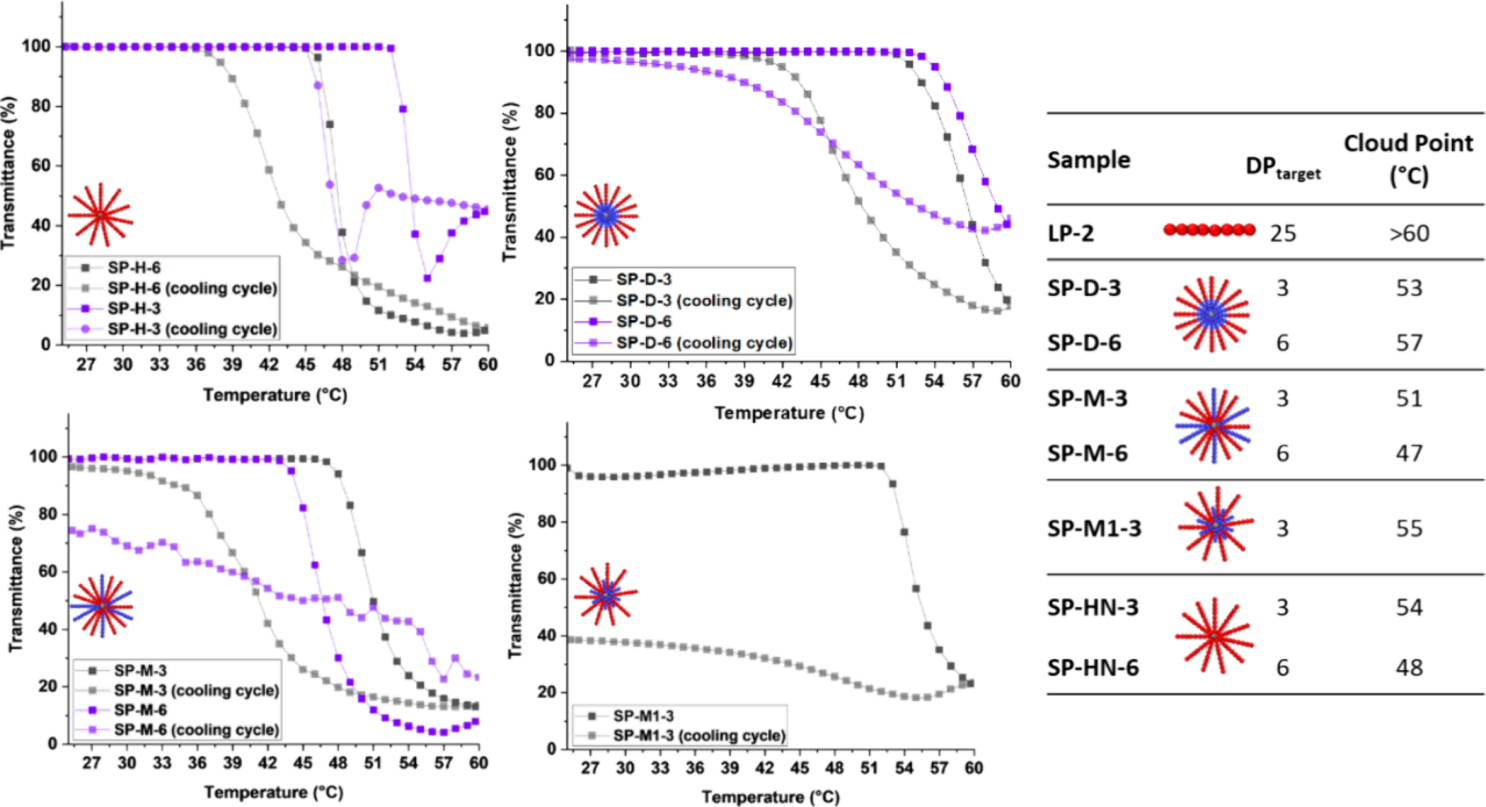
Turbidity measurements for star copolymers and linear polymer LP-2, UV–vis transmittance at 633 nm (1 cycle, 1 °C/min from 25 °C–60 °C). Table with cloud points = 50% transmittance.

The eight deprotected star polymers all showed an LCST transition (Figure 7), however, for all the polymers, the cloud point was far above 37 °C, the physiological temperature at which their biological activity is determined. Therefore, no interference due to aggregation is expected when determining their antimicrobial activity or toxicity at this temperature. However, it was observed that the LCST behavior changes in regard to the molecular weight and chemical composition of the polymers. For the pNIPAm stars SP-H-3 and SP-H-6, the transmission decreases in a sharp manner, with a drop from 100 to 20% transmission within a temperature change of 4 °C. For SP-H-3, a sharp increase in transmission is observed around 55 °C, which could be related to the polymer fully crashing out of solution, thereby increasing transmittance.

As a direct comparison to SP-H-6, the polymer SP-M-3, with a similar molecular weight and number of arms, decreases to 20% transmission within a temperature change of 9 °C. This indicates that the addition of cationic pAEAm_25_ arms both raises the LCST and decreases the aggregation kinetics. Finally, the samples were cooled to 25 °C to determine if the phase separation and aggregation were reversible. This was the case for the SP-H and SP-D polymers, as well as SP-M-3. However, SP-M-6 and SP-M1–6 did not appear to redissolve and crashed out fully during the heating cycle. This irreversible aggregation could be attributed to the overall higher number of pNIPAm arms within these two miktoarm stars.

**Figure 8:**
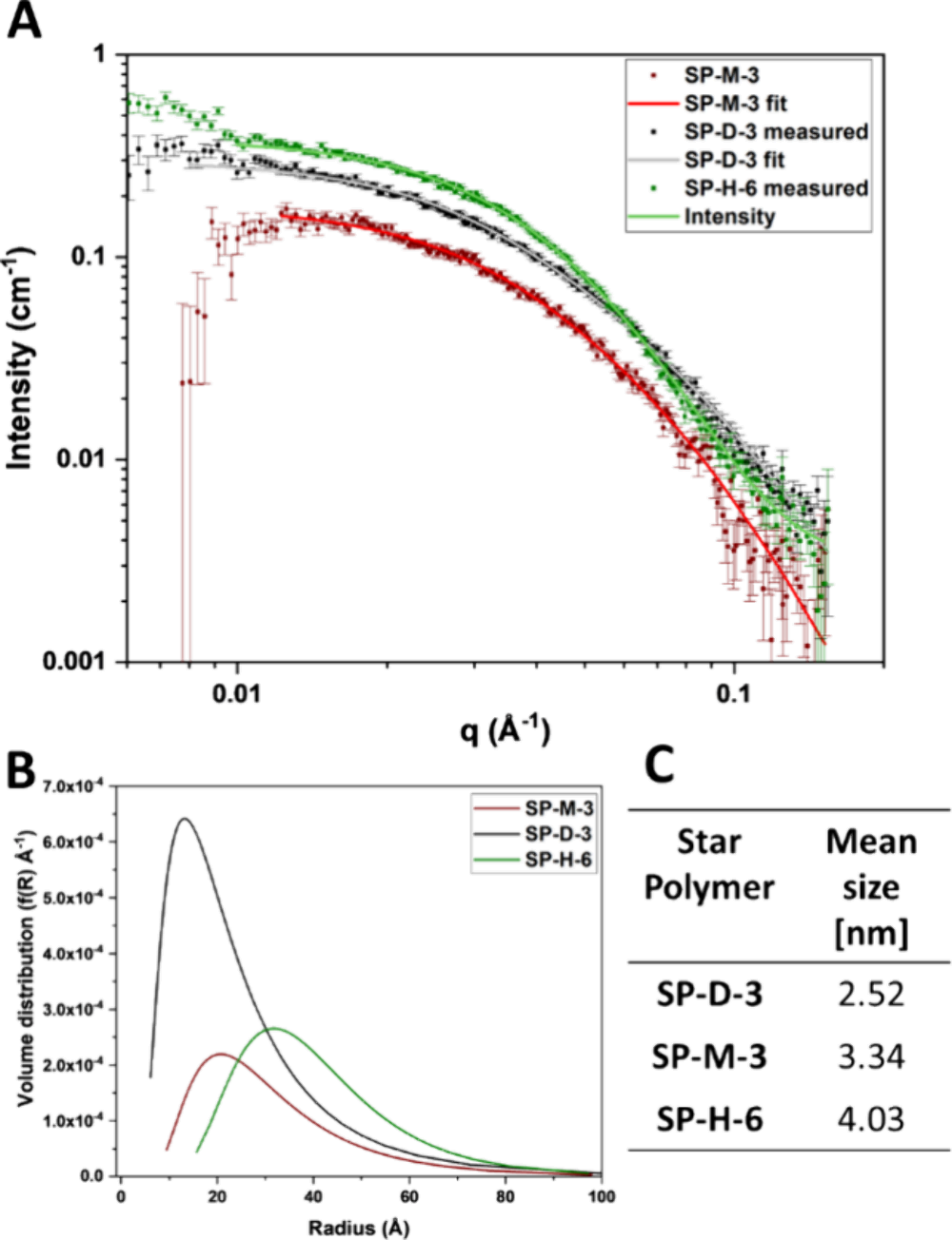
SAXS measurements of star polymers SP-M-3, SP-D-3, and SP-H-6. (A) SAXS scattering profile of measured data and fit. (B) Volume distribution of radii. (C) Mean size of polymers.

In order to further investigate the behavior of the star polymers in solution, small-angle light scattering (SAXS) (Figure 8) was performed in PBS. In order to obtain sufficient scattering intensities, the star polymers were dissolved at a concentration of 10 mg mL^–1^. Therefore, it should be noted that these results cannot replicate the behavior in solution of these polymers in our biological assays, which are conducted at a maximal concentration of 1 mg mL^–1^. Nevertheless, the direct comparison of diblock vs miktoarm stars might give some insights into the potential influence of the location of cationic units on their behavior in solution. SP-M1-3 could not be dissolved at this concentration, as well as the stars with higher numbers of arms (SP-D-6/SP-M-6). In order to compare the influence of cationic units on behavior in solution, the pNIPAm homopolymer star SP-H-6 was also measured, as its molecular weight is comparable to that of the cationic stars, due to the overall smaller size of the homopolymer stars.

The SAXS data was fitted as spheres, with the radius given by a log-normal distribution. Even at this high concentration, the signal overall was quite weak. The scattering results (Figure 8A) indicate that the miktoarm star scattering intensity is slightly lower compared to the other two stars at the same concentration, possibly due to the higher exposure of cationic units on the outer side of the star structure. However, no other differences in the scattering patterns can be observed. Furthermore, the mean diameter of the CSS polymers obtained through this fit is similar to the hydrodynamic radius obtained by SEC of the protected polymers, except for SP-D-3, where the mean size is slightly smaller than the hydrodynamic radius (3.66), possibly due to solvent effects of DMF versus PBS.

#### In Vitro Analysis of CCS Polymer Antimicrobial Properties

In order to assess the antimicrobial activity of these core-cross-linked stars against bacterial cells, the MIC of each compound was determined using the broth microdilution method against two bacterial species (also used in our previous study^8^) as representative models: the Gram-negative strain *P. aeruginosa* PA14, a highly virulent strain, and *S. aureus* USA300, a methicillin-resistant strain, both of which are strong biofilm formers. The results are reported in a weight-based concentration and a molar concentration so as to compare MIC values relative to and independent of molecular weight. Both are reported below, along with a direct comparison to the 4-arm star D-SP25 (A-N) from our previous study. For *P. aeruginosa* PA14, no activity was observed for any of the CCS polymers, which was very surprising given that the 4-arm stars in our previous study exhibited significant activity toward this strain, while being inactive against *S. aureus* USA300.^8^ It seems that, with an increased number of arms, these polymers lose their activity, regardless of cationic unit positioning, possibly due to the two lipid bilayers within the membrane.

However, all CCS polymers showed activity toward *S. aureus* USA300, except for SP-D-6. Both SP-D-3 and SP-M1-3 showed inhibition at the highest measured concentration against this strain, and the highest overall activity was observed for the SP-M-3 and SP-M-6 stars. The same trends are observed when looking at the micromolar values (Figure S5); however, when comparing micromolar MIC values, SP-M1-3 is slightly more active compared to SP-D-3. It seems that increasing the number of arms from 4 to 16 slightly raised activity toward *S. aureus* when comparing D-SP25 (A-N) to SP-D-3. However, upon further increase of arm numbers to 29, the star loses activity again (Table 2).

**Table 2:**
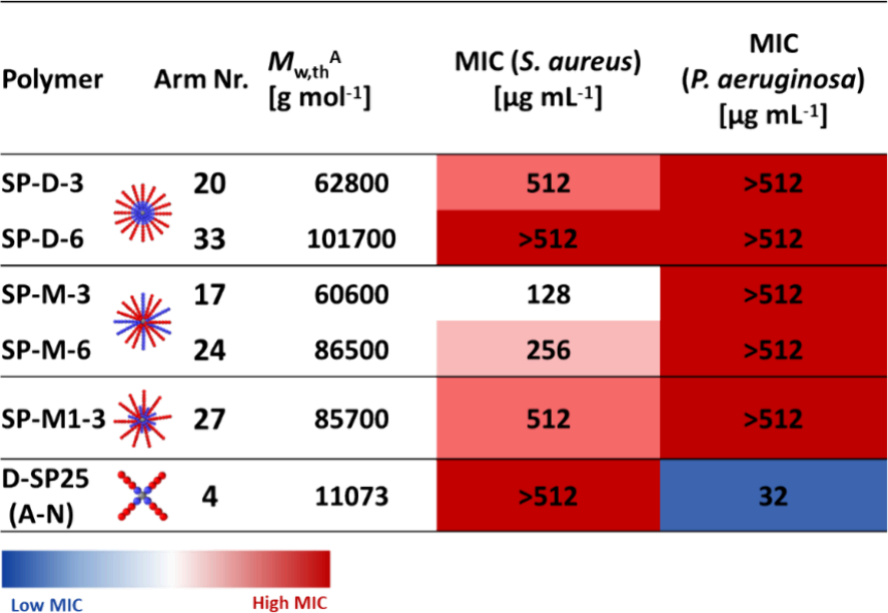
Antimicrobial Activity of the Copolymers^b^

a Theoretical molecular weight of deprotected star copolymers calculated based on the average number of arms of protected star polymers and theoretical weight of the deprotected linear mCTA.

b MIC values expressed in (μg mL^–1^) and (μM) of the copolymers tested in caMHB against *S. aureus* USA300 and *P. aeruginosa* PA14 with a concentration range of (4–512 μg mL^–1^). The heatmap shows high activity/low MIC in blue and low activity/high MIC in red. Three independent biological experiments (performed on different days) were performed.

The overall lowered activity of these diblock star polymers toward *S. aureus* could be due to the pNIPAm block shielding the cationic units from binding to the peptidoglycan layer.^26^ The same hypothesis can be made for the miktoarm star SP-M1-3 with shorter cationic arms. This argument is further supported by the highest measured activity against *S. aureus* observed for the miktoarm stars with equally long cationic and apolar arms SP-M-3/6, wherein the cationic units are likely more exposed to the bacterial membrane, leading to an increased interaction. Furthermore, as the arm numbers are increased from 4 to 20 and higher, electrostatic interactions between the cationic arms may play a role, resulting in the “fanning” out of the homopolymeric cationic arms in the miktoarm structures, enhancing binding to the membranes. However, for the diblock stars, even if such repulsion were to potentially occur within the core, it did not appear to enhance activity.

In order to compare the CCS polymers to the 4-arm star, the structural difference due to the location of the RAFT agent R-group (carboxylic acid group) within the star architecture has to be assessed. In the 4-arm star polymers, the synthesis strategy resulted in the RAFT carboxylic acid group forming the cross-linked core from which the arms are grown, while in the arm-first method, the RAFT carboxylic acid group is located on the outer side of the star structure. As carboxylic acid groups are acidic with a p*K*_a_ value between 3 and 5, they will be mostly deprotonated at pH 7, at which these in vitro assays are performed and therefore negatively charged. This could influence the antimicrobial activity of these structures by counteracting the overall cationic charges. Furthermore, this may explain the low charge densities observed in the DLS measurements. This, in addition to reduced exposure of the AEAm units to the membrane, could therefore explain the overall low activity observed for the diblock stars. This negative charge could also be responsible for the complete loss of antimicrobial activity toward *P. aeruginosa* in comparison to the 4-arm stars, as perhaps the negative charges resulted in decreased ability of these polymers to bind to and penetrate the two lipid bilayer membranes in Gram-negative bacteria. However, to further examine this hypothesis, the polymers would need to be tested against further Gram-positive and -negative bacteria strains to determine the cause of this inversed selectivity toward the Gram-positive strain compared to our previous study.

#### In Vitro Analysis of Hemocompatibility

To determine the compatibility of these compounds with mammalian cells, the hemolytic activity and hemagglutination were determined through the incubation of the polymers at a concentration range of 4–512 μg mL^–1^ with sheep red blood cells (Table 3).

**Table 3:**
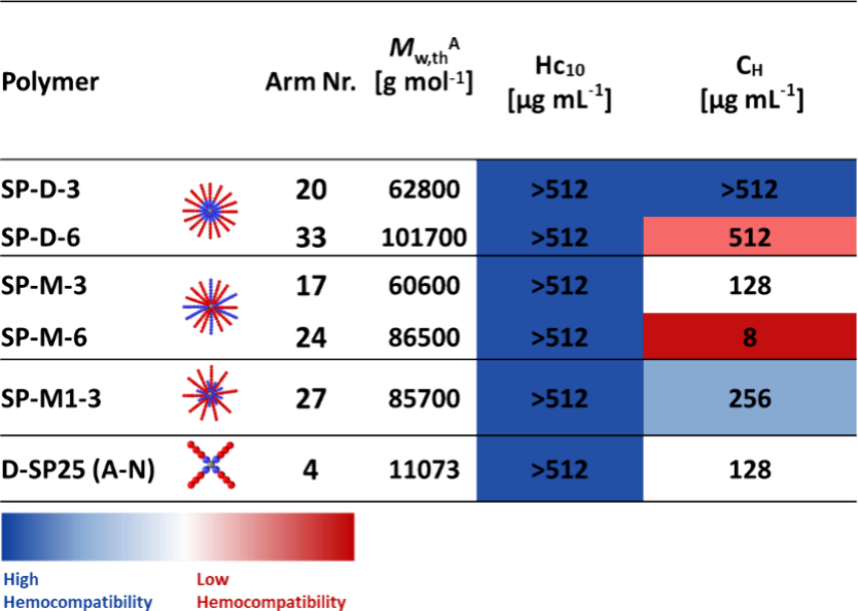
Hemolytic Activity (HC_10_ – Minimum Concentration Which Induces Lysis of 10% of Red Blood Cells) and Hemagglutination (C_H_ – Minimum Concentration of the Antimicrobial Agent or Treatment at Which 10% of Red Blood Cells Aggregated) Expressed in (μg mL^–1^) and (μM)^b^

a Theoretical molecular weight of deprotected star copolymers calculated based on the average number of arms of protected star polymers and theoretical weight of the deprotected linear mCTA.

b Three independent biological experiments (performed on different days) were performed, and the highest toxicity value was reported.

None of the star polymers induced hemolytic activity; however, all polymers, except for SP-D-3, caused hemagglutination. SP-M-6 aggregated red blood cells at the lowest overall concentration, while SP-M-3 and SP-M1-3 aggregated at 16/32 times higher weight concentration, respectively. When considering the micromolar values, the trends are the same (Figure S6) with SP-M-3 and SP-M1-3 aggregated at 21/30 times higher molar concentration, respectively.

Positioning the cationic units in the center of the star structure appears to suppress aggregation behavior, but this increases gradually with increasing length and number of the cationic arms in the miktoarm stars. In correlation with antimicrobial activity, it seems that cationic units are the driving factor for increased hemagglutination, which is in line with our previous study on 4-arm stars.^8^ When comparing to the 4-arm star D-SP25 (A-N), it seems the increased number of arms has decreased the aggregation for the diblock stars. For D-SP25 (A-N) and SP-M-3, the same weight-based concentration for aggregation is observed; however, when comparing micromolar concentrations, SP-M-3 aggregates at a lower concentration. Furthermore, the MIC for the star SP-M-3, with the highest antimicrobial activity, is the same as the concentration at which hemagglutination is observed; therefore, these polymers are not selective toward bacterial cells when using the hemagglutination values to determine selectivity.

Overall, it seems that these CCS polymers are inferior in their biological activity compared to our most promising compounds reported before.^8^ However, we were able to further demonstrate the importance of cationic unit exposure to the bacterial membrane, as the increased exposure of cationic units in the miktoarm star polymers appears to result in increased antimicrobial activity. Furthermore, the successfully optimized synthesis of CCS stars could be used in future work to investigate further structural parameters such as arm length and cationic ratio within the arms.

## Conclusions

The arm-first synthesis strategy was successfully used to synthesize a library of amphiphilic cationic core-cross-linked star polymers via RAFT polymerization, with both diblock and homopolymer arms, in an efficient and straightforward manner. All polymers exhibited LCST behavior, with the cloud point raised by more than 10 °C above the LCST observed in the literature for linear pNIPAm,^20^ showing that the star architecture can alter thermoresponsive properties. The antimicrobial activity was increased for these structures when switching from a diblock to a miktoarm CCS, indicating that the exposure of cationic units to the membrane is essential to obtain active polymers. Furthermore, while all synthesized polymers displayed no hemolytic activity in sheep red blood cells, there was a noted increased tendency to aggregate red blood cells with increased exposure of cationic units. To conclude, the increased number of arms did not increase antimicrobial activity in direct comparison to the 4-arm s-SNAPs; however, we demonstrated an efficient method to synthesize both diblock and miktoarm stars, which could be further improved through structural modifications. It may be that a “sweet spot” in terms of arm number lies between the two extremes. This could be further investigated in future work, for example, through increasing the length of arms within this system, which has been shown to reduce the arm number when synthesizing star polymers via the “arm-first” method^27^ and may lead to higher exposure of cationic units from the core in diblock CCS.

## Supporting Information Available

^1^H NMR spectra of all compounds synthesized in this work, SEC traces of all star and linear polymers, MIC, hemolytic activity and hemagglutination data expressed in μM, and additional information on polymer synthesis conditions (PDF)
