# Supplementary material for: Cationic Star Polymers Obtained by the Arm-First Approach—Influence of Arm Number and Positioning of Cationic Units on Antimicrobial Activity
Source: Biomacromolecules. 2024 Dec 2;26(1):190–200. doi: 10.1021/acs.biomac.4c00882 (PMC11733951; doi:10.1021/acs.biomac.4c00882)
Supplement: Supplementary file 1 — bm4c00882_si_001.pdf [file bm4c00882_si_001.pdf]

Supplementary Information

Cationic Star Polymers obtained by the arm first approach – Influence of arm number and positioning of cationic units on antimicrobial activity

Sophie Laroque[a], Katherine E. S. Locock[b], Sébastien Perrier[a,c,d] *

[a] *Department of Chemistry, University of Warwick, Gibbet Hill Road, Coventry CV4 7AL, UK.*

[b] *CSIRO Manufacturing, Clayton, Victoria 3168, Australia*

[c] *Division of Biomedical Science, Warwick Medical School, University of Warwick, Coventry, CV4 7AL, UK*

[d] *Faculty of Pharmacy and Pharmaceutical Sciences, Monash University, 381 Royal Parade, Parkville, VIC 3052, Australia*

* Corresponding author: s.perrier@warwick.ac.uk

Table S1: Reactions conditions for the RAFT polymerization of the linear (co)polymers.

**Linear Polymer**	**LP-1A**	**LP-1B**	**LP-2**	**LP-3**	**LP-4**
**Monomer 1**	NIPAm	-	NIPAm	-	
**Monomer 2**	-	BocAEAm	-	BocAEAm	BocAEAm
**$DP_{target\ total}$**	25	25	25	25	12.5
**$DP_{target\ Monomer\ 1}$**	17.5	-	25	-	-
**$DP_{target\ Monomer\ 2}$**	-	7.5	-	25	12.5
**$m_{monomer1,added}$ (mg)**	1697	-	1697	-	-
**$m_{monomer2,added}$ (mg)**	-	1337	-	535	535
**$m_{CTA,added}$ (mg)**	204.3	1901.7 (LP-1A)	143	23.8	47.7
**$m_{Initiator,added}$ (mg)**	6	8	4.2	0.7	1.4
**$V_{dioxane,added}$ (mL)**	5	5	5	5	5
**$V_{water,added}$ (mL)**	-	-	-	-	-
**V_{total} (mL)**	5	10	5	5	5
**$[CTA]_0/[I]_0$**	40	30	40	40	40

Table S2: Reactions conditions for the RAFT polymerization of the CSS (co)polymers.

**CSS Polymer**	**SP-D-3**	**SP-D-6**	**SP-M-3**	**SP-M-6**	**SP-M1-3**	**SP-M1-6**
**Monomer (CL)**	MBAm	MBAm	MBAm	MBAm	MBAm	
**$DP_{target\ total}$**	3	6	3	6	3	6
**$m_{monomer1,added}$ (mg)**	62	62	62	62	62	62
**$m_{mCTA\ 1}$**	LP-1	LP-1	LP-2	LP-2	LP-2	LP-2
**$m_{mCTA\ 2}$**	-	-	LP-3	LP-3	LP-4	LP-4
**$m_{mCTA\ 1,added}$ (mg)**<br>**$m_{mCTA\ 2,added}$ (mg)**	510<br>-	255<br>-	286<br>224	143<br>112	204<br>201	102<br>101
**$m_{Initiator,added}$ (mg)**	1.4	0.7	1.4	0.7	1.4	0.7
**$V_{dioxane,added}$ (µL)**<br>**$V_{water,added}$ (µL)**	1600<br>400	1600<br>400	1600<br>400	1600<br>400	1600<br>400	1600<br>400
**V_{total} (mL)**	2	2	2	2	2	2
**$[CTA]_0/[I]_0$**	20	20	30	30	30	30

Table S3: Reactions conditions for the RAFT polymerization of the CSS homopolymers.

**CSS Polymer**	**SP-H-3**	**SP-H-6**
**Monomer (CL)**	MBAm	MBAm
**$DP_{target\ total}$**	3	6
**$m_{monomer1,added}$ (mg)**	62	62
**$m_{mCTA\ 1}$**	LP-2	LP-2
**$m_{mCTA\ 2}$**	-	-
**$m_{mCTA\ 1,added}$ (mg)**<br>**$m_{mCTA\ 2,added}$ (mg)**	408	143
**$m_{Initiator,added}$ (mg)**	1.4	0.7
**$V_{dioxane,added}$ (µL)**<br>**$V_{water,added}$ (µL)**	1600<br>400	1600<br>400
**V_{total} (mL)**	2	2
**$[CTA]_0/[I]_0$**	30	30

Table S4: Kinetic Reaction of CCS polymer Synthesis – Values from ^{1}H-NMR and SEC Analysis.

**Time (min)**	**Conversion crosslinker (^{1}H-NMR) (%)**	**Arm incorporation (GPC) (%)**	**Mn (g mol^{-1}) (Star Peak)**	**Đ (Star Peak)**
**5**	3	20	5500	1.35
**15**	43	41	13000	1.09
**30**	79	68	15000	1.16
**45**	99	77	16500	1.18
**60**	99	80	16900	1.19
**120**	99	84	17700	1.2

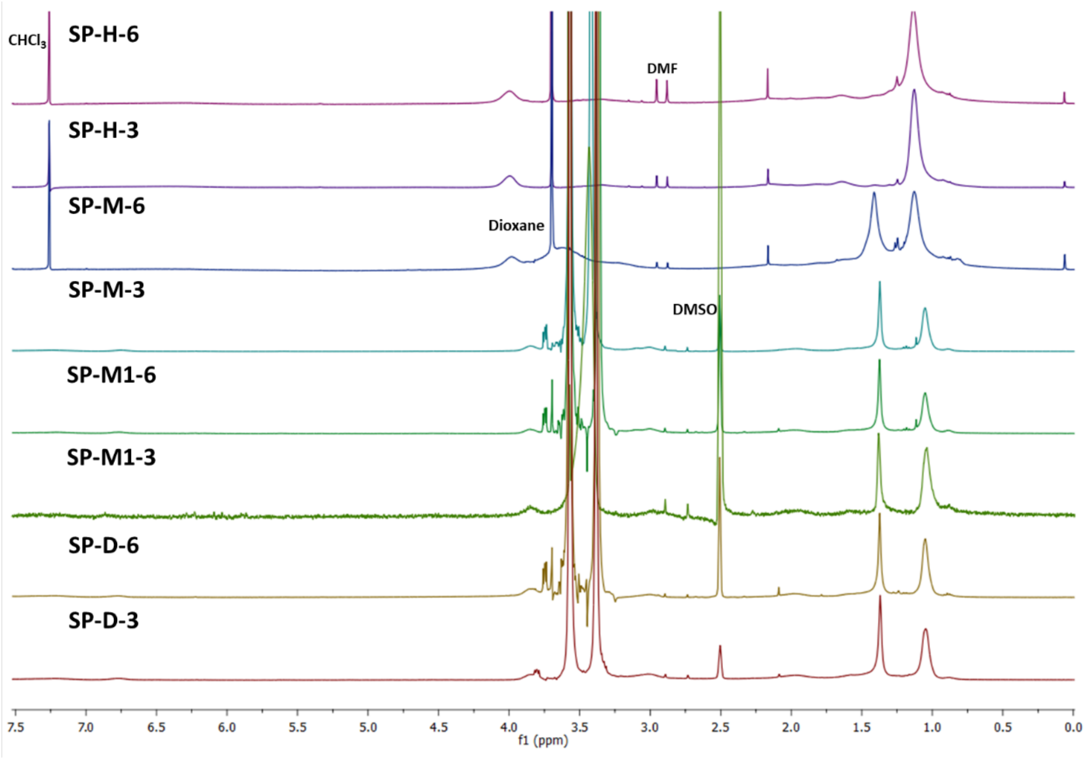

Figure S1: ^{1}H-NMR spectra of 6 Boc-protected star polymers and Homopolymer stars in $DMSO-d_6/CDCl_3$.

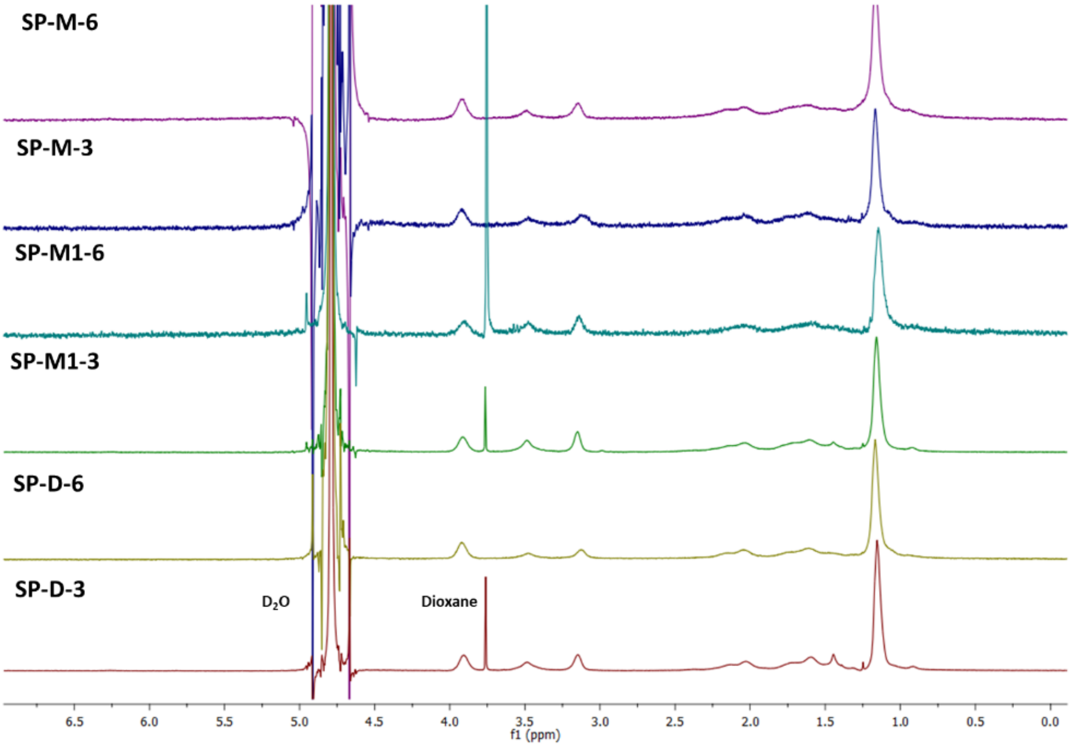

Figure S2: ^{1}H-NMR spectra of 6 deprotected cationic star polymers (pre-dialysis) in D_2O.

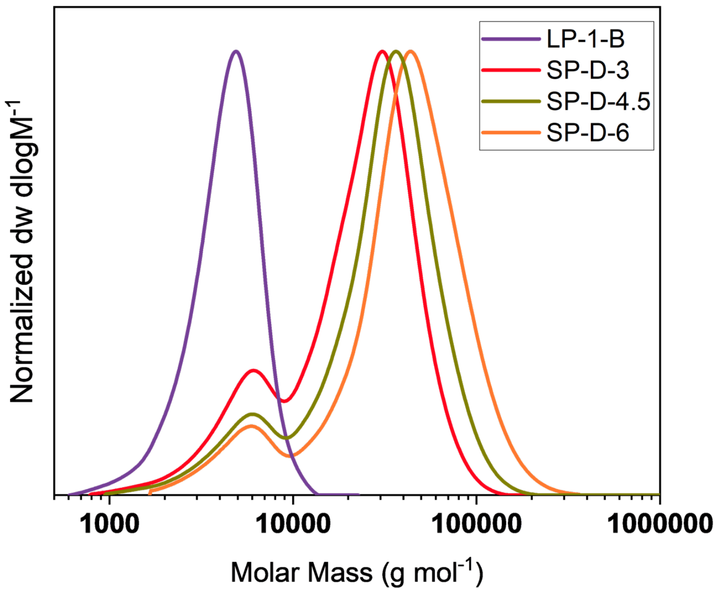

Figure S3: SEC traces of Diblock CCS with increasing CL/mCTA ratio.

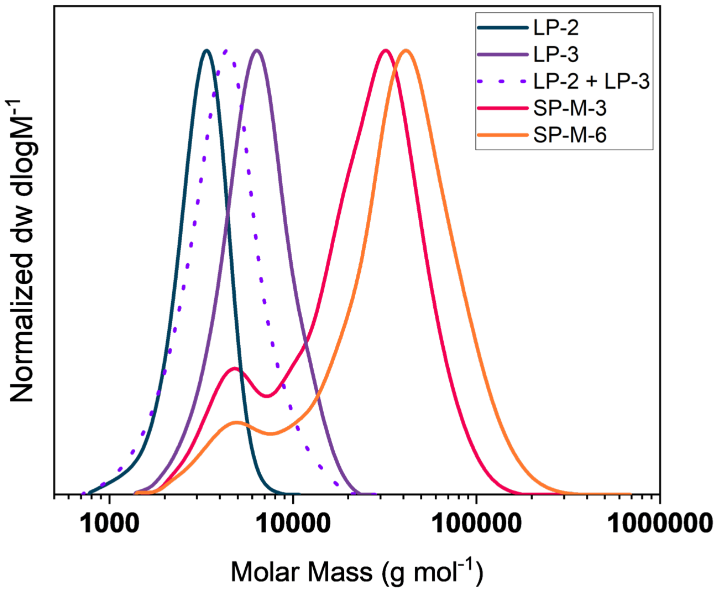

Figure S4: SEC traces of Linear Polymers LP-2 and LP-3 mixed in a 70/30 ratio compared to individual SEC traces of linear polymer and leftover trace of unconsumed linear polymer.

Polymer	Arm Nr.	$M_{w,th}$[A] [g mol^{-1}]	MIC (*S. aureus*) [µM]	MIC (*P. aeruginosa*) [µM]
SP-D-3	20	62800	8.2	>8.2
SP-D-6	33	101700	>5	>5
SP-M-3	17	60600	2.1	>8.4
SP-M-6	24	86500	3	>5.9
SP-M1-3	27	85700	6	>6
D-SP25 (A-N)	4	11073	>46.2	2.9

Low MIC   High MIC

Figure S5: Antimicrobial activity of the copolymers. MICs values expressed in (µM) of the copolymers tested in caMHB against *S. aureus* USA300 and *P. aeruginosa* PA14.

Polymer	Arm Nr.	$M_{w,th}$[A] [g mol^{-1}]	Hc_{10} [µM]	C_H [µM]
SP-D-3	20	62800	>8.2	8.2
SP-D-6	33	101700	>5	5
SP-M-3	17	60600	>7.5	2.1
SP-M-6	24	86500	>6	0.1
SP-M1-3	27	85700	>6	3
D-SP25 (A-N)	4	11073	>46.2	11.6

High Hemocompatibility   Low Hemocompatibility

Figure S6: Hemolytic activity (Hc_{10}) and hemagglutination (C_H) of the copolymers expressed in (µM) of the copolymers.
